# No Evidence That Gratitude Enhances Neural Performance Monitoring or Conflict-Driven Control

**DOI:** 10.1371/journal.pone.0143312

**Published:** 2015-12-03

**Authors:** Blair Saunders, Frank F. H. He, Michael Inzlicht

**Affiliations:** 1 Department of Psychology, University of Toronto, Toronto, Canada; 2 Rotman School of Management, University of Toronto, Toronto, Canada; Radboud University Nijmegen, NETHERLANDS

## Abstract

It has recently been suggested that gratitude can benefit self-regulation by reducing impulsivity during economic decision making. We tested if comparable benefits of gratitude are observed for neural performance monitoring and conflict-driven self-control. In a pre-post design, 61 participants were randomly assigned to either a gratitude or happiness condition, and then performed a pre-induction flanker task. Subsequently, participants recalled an autobiographical event where they had felt grateful or happy, followed by a post-induction flanker task. Despite closely following existing protocols, participants in the gratitude condition did not report elevated gratefulness compared to the happy group. In regard to self-control, we found no association between gratitude—operationalized by experimental condition or as a continuous predictor—and any control metric, including flanker interference, post-error adjustments, or neural monitoring (the error-related negativity, ERN). Thus, while gratitude might increase economic patience, such benefits may not generalize to conflict-driven control processes.

## Introduction

Self-control comprises effortful, goal-directed processes that help to restrain automatic impulses and response tendencies. Recently, DeSteno, Li, Dickens and Lerner [1] explored a less effortful route to goal attainment, proposing that gratitude promoted economic patience during financial decision making. Identified as a positive moral emotion, gratitude typically arises in a social context when someone gives—or attempts to give—something valuable to another person [2, 3]. On a functional level, gratitude putatively facilitates reciprocity and altruism, where a person is more likely to engage effort or make personal sacrifices if they feel gratitude towards a benefactor [1–3]. Indeed, feelings of gratitude have been associated with increased effort during reciprocal interactions [2], facilitating long-term interpersonal gains even when such labor comes at some sacrifice or cost to the individual [3]. What is intriguing about the findings of Desteno et al. [1], however, is that gratitude reduced the tendency to discount future rewards in a financial domain relatively devoid of explicit interpersonal context. These findings suggest that the adaptive benefits of gratitude might generalize across self-regulation settings, perhaps by inhibiting the influence of more automatic, impatient processes that generate self-control failure [1, 4, 5]. In the current study we further tested the hypothesis that gratitude improves self-control, testing if feelings of gratitude accompany enhanced neural performance monitoring and conflict-driven control.

### Does gratitude facilitate self-control more broadly?

Temporal discounting has long served as a measure of impulse control [6, 7]. Furthermore, measures derived from laboratory delay discounting tasks predict a remarkable array of real-world phenomena. In one classic longitudinal study, for example, the ability to resist immediate gratification in children aged 4 years predicted a number of metrics of wellbeing and social competence when this same cohort reached adolescence [7]. Therefore, the suggestion that gratitude facilitates patience in such tasks might have important implications for everyday self-regulation. In addition to temporal discounting, however, decades of control research have developed a number of laboratory paradigms that test the ability to monitor for and regulate conflicting impulses and response tendencies. Expanding on recent suggestions [1], we investigated the relationship between gratitude and established behavioural and neurophysiological metrics of conflict-driven control.

Self-control is commonly assessed using conflict paradigms. In the Eriksen flanker task [8], for example, a central target letter is surrounded by compatible (e.g., SSSSS) or incompatible (e.g., HHSHH) flanker letters. On conflict trials participants must override the incorrect response primed by the incompatible flanker letters, leading to considerable behavioural interference relative to compatible trials [9]. In addition to these conflict effects, errors often occur when responses are issued hurriedly, without sufficient stimulus evaluation [10]. As such, another important feature of control is to rapidly evaluate self-control failures and flexibly issue remedial adjustments to avoid their re-occurrence. For example, responses often slow after mistakes [11], an effect commonly attributed to a strategic increase in post-error caution [9]. Mirroring suggestions that lab-based measures of temporal discounting predict everyday self-regulation, both conflict control and error adjustments are associated with several real world phenomena, including trait impulsivity [12], daily stress regulation [13], and academic attainment [14]. Following recent suggestions that gratitude facilitates self-regulation, we tested if this emotion is associated with improved conflict control and post-error recovery.

In addition to these behavioural metrics, we also assessed the impact of gratitude on the neurophysiological processes that evaluate the present need for control. The medial prefrontal cortex is implicated in performance monitoring [9], and the error-related negativity (ERN) is one commonly studied event-related potential (ERP) related to this monitoring process [15, 16]. The ERN is a frontocentral ERP that peaks within 100 ms after mistakes, and, besides differentiating correct actions from mistakes, the ERN also reflects the affective or motivational significance of errors [17–20] and is reduced as a function of impulsive traits and psychopathologies associated with maladaptive reward seeking [21, 22]. Thus, if gratitude lessens the need for immediate gratification, we also predicted that this emotion will be associated with enhanced neural monitoring.

## Method

### Participants

A priori we determined to stop collecting data once we had recruited at least 60 participants for the experiment; this sample size was determined based on the number of participants contributing to each experimental cell in a previous study of gratitude [1]. Our pre-registered hypotheses, experimental materials, and data-sets are freely available on the Open Science Framework (osf.io/mtrys). 61 undergraduate students from the University of Toronto Scarborough participated in return for course credit. Three participants were excluded from these analyses because essay responses were not recorded for these individuals. This left us with a sample of 58 participants (32 females; mean age = 18.4, *SD* = 1.4 years). Participants were informed that the purpose of the current study was to investigate the effects of memory recall on reaction time performance. As with prior studies, we used this cover story to avoid informing participants that the main purpose of the study was to investigate the effects of emotion on performance. Our research had human subject approval from the University of Toronto Research Ethics Board, and written informed consent was obtained from all participants

### Task and Procedure

**Fig 1:**
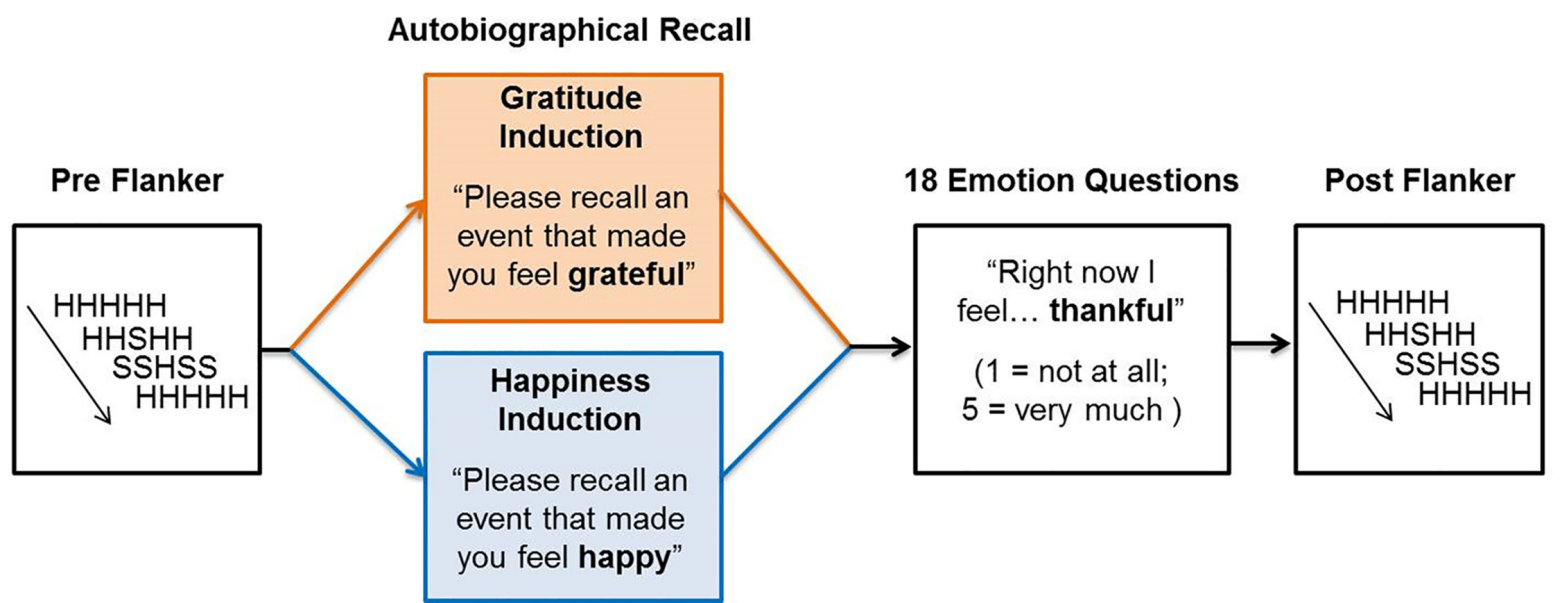
An illustration of the experimental procedure.

Following established procedures [1], we induced gratitude and a control emotion (happiness) using an autobiographical recall task. Our experiment used a mixed design where participants in each induction group contributed data to both pre-induction and post-induction behavioural and ERP analyses (see Fig 1 for an illustration of the overall procedure). Therefore, each group’s pre-induction performance provided baseline measures of control (i.e., behavioural performance and ERPs), with the experimental effects of our induction procedures assessed through comparison with the equivalent post-induction levels of the same measures. Thus, rather than including a separate neutral control group, each participant acted as his/her own “control” in the current study. This pre-post protocol was utilized largely to reduce the levels of between-subject error variance, increasing statistical power by reducing the influence of inter-individual variation. Therefore, a specific effect of gratitude on a given variable would be determined if gratitude had a specific effect on performance (compared to baseline) that did not occur for participants in the happy condition.

Immediately after fitting the EEG apparatus, all participants performed a pre-induction flanker task. Participants identified the central letter in a five letter string. All flanker arrays in the current study were five letters long, consisting of the letters H and S. Participants were instructed to press the Z key on their keyboard if the target was S and the / key if the target was H. Manual responses were recorded using a millisecond accurate DirectIN keyboard (Empirisoft, New York, NY), with key assignment in the QWERTY format. Compatible and incompatible stimulus arrays were formed by altering the relationship between the flanker letters and the target stimuli. For compatible arrays, the flanker letters matched the target (e.g., HHHHH). In contrast, for the incompatible arrays, the flankers primed the opposite response to the target (e.g., SSHSS).

Trials commenced with a central fixation cross (250 ms). Subsequently, the flanker letters appeared onscreen 100 ms prior to the onset of the central target stimuli. This stimulus-onset asynchrony was used to increase the amount of response priming caused by the flanker stimuli relative to the target. Targets were presented until response or for a maximum of 1500 ms, followed by a blank screen for 1000 ms. Participants first completed 20 practice trials, followed by 500 experimental trials divided equally into 5 blocks. Compatible and incompatible flanker trials were presented with equal probability, and participants were allowed to take short, self-paced breaks between blocks.

Immediately following the flanker task, participants performed an autobiographical recall task that aimed to induce either feelings of happiness or gratitude. The methods for these induction procedures aimed to closely replicate those used by DeSteno et al. [1] Participants were presented with the following instructions: “Please recall an event that made you feel grateful (or happy). You have 5 minutes to write your response, so try to include as much detail as possible.” Participants were then allowed to type their essay responses directly into a field that appeared on-screen, and could not move on to the next part of the experiment before the full 5 minutes had elapsed. The participants then answered a questionnaire assessing their current emotional state to act as a manipulations check. The affective descriptors used to assess gratitude were *grateful*, *thankful*, and *appreciative*, (cronbach’s α = .90) and the happiness words were *happy*, *content*, and *pleasant* (cronbach’s α = .83). These scales were embedded in a series of filler descriptors (*sad*, *angry*, *annoyed*, *bored*, *accomplished*, *confident*, *proud*, *negative*, *anxious*, *frustrated*, *guilty* and *good*). All questions were framed to provide a state measure of emotion (e.g., “Right now I feel thankful”), participants answered using a 5-point Likert scale (1 = not at all; 5 = very much), and question order was randomized between participants.

Participants then completed a post-induction flanker task that followed a procedure identical to the pre-induction task.

### Electroencephalographic (EEG) recording

EEG activity was recorded from 11 Ag/AgCl sintered electrodes embedded in a stretch-lycra cap (Electro-cap International, Inc., Eaton, Ohio). The montage consisted of midline electrodes (FPz, Fz, FCz, Cz, CPz, Pz, & Oz), referenced to the average of bilateral earlobe electrodes. Vertical electro-oculography was measured using two electrodes placed above and below the right eye. Four additional electrodes measured electromyographic signals from over facial musculature (*corrugator supercilii* and *zygomaticus major*), however, this was not analysed for present purposes.

The continuous EEG was digitized using a sample rate of 1024 Hz and electrode impedances were maintained below 5 KΩ using ANT acquisition hardware (Advanced Neuro Technology, Enschede, the Netherlands). Offline, data was analysed using Brain Vision Analyser software (v.2) (Brain Products, GmbH, Gilching, Germany). The data was first band-pass filtered (0.1 to 20 Hz) and ocular correction was achieved using regression based procedures [23]. Subsequently, an automatic procedure was employed to detect and remove EEG artifacts. The criteria applied were a voltage step of more than 15 μV between sample points, a voltage difference of 150 μV within 150 ms intervals, voltages above 85 μV and below -85 μV, and, finally, low activity was identified if the voltage difference between the maximum (i.e., most positive) and minimum (i.e., most negative) sample point for a 100 ms interval was less than 1 μV.

ERP epochs commenced 200 ms prior to each response and lasted for 1200 ms. The ERPs were then corrected using a 100 ms baseline that commenced 150 ms before the response, and averaged within participants independently for correct and incorrect trials. The ERN and its correct trial equivalent ERP (the correct related negativity, CRN) were operationalized using a peak-to-peak measurement where the negative maxima 0–120 ms after the response was subtracted from the most positive potential -100 to 0 ms before the response. Some authors advocate using mean amplitude rather than peak measures to operationalize ERPs [24]. In our results, all patterns of significant effects were identical when we used mean amplitude 0 to 100 ms following the response to quantify the ERN and CRN rather than the peak-to-peak approach.

### Statistical analysis

Multilevel models were used to analyse all questionnaire, behavioural, and ERP data using the MIXED function in SPSS (v. 22). In each MLM we modelled a random intercept for each participant using an unstructured correlation metric. Effects were determined to be robust if the 95% confidence intervals for an effect did not span zero [25]. Semi-partial R^2^ (R^2^_β_) is reported as an effect size [26]. Mean centering was used whenever a variable was used as a continuous predictor or covariate. In any covariate models, we included self-reported gratitude and happiness as covariates simultaneously to test if gratitude predicted our dependent variables independently from general positive affect (i.e., happiness). However, it should be noted that we found no novel significant effects in any behavioral or ERP analyses when gratitude was used as a predictor without controlling for happiness.

## Results

### Manipulations check

We first conducted a manipulations check on the self-reported emotion ratings of gratitude (overall *M* = 3.47; *SD* = 1.26; *range*: 1 to 5) and happiness (overall *M* = 3.24; *SD* = 1.03; *range*: 1 to 5). In line with previous studies of gratitude [1], we made the *a priori* prediction that groups would not differ on positive affect in general (i.e., happiness), but rather that participants who recalled a grateful autobiographical event would report higher gratefulness than participants who recalled a happy memory. Initially, we tested if happiness levels differed between groups (happy = -1; grateful = 1). In line with our expectations, participants reported similar levels of happiness in the happy (*M* = 3.22; *SE* = 0.10) and grateful (*M* = 3.25; *SE* = 0.10) condition (*b* = -.03, *SE* = 0.14), 95% CIs [-0.30, 0.23], R^2^_β_ < .001.

We then tested if gratitude levels differed between groups while controlling for overall positive emotions (i.e., adding happiness as a covariate, see [1]). Contrary to our predictions, self-reported gratitude levels did not differ between groups (*b* = -0.03, *SE* = 0.13), 95% CIs [-0.28, 0.22], R^2^_β_ < .001, while self-reported happiness was positively associated with gratitude scores (*b* = 0.77, *SE* = 0.06), 95% CIs [0.65, 0.89], R^2^_β_ = .40 in this model. Finally, we tested for differences in self-reported gratitude between conditions without controlling for self-reported happiness. Here, we found no difference between gratitude levels in the happy (*M* = 3.44; *SE* = 0.12) and the grateful (*M* = 3.49; *SE* = 0.12) conditions, (*b* = -0.06, *SE* = 0.16), 95% CIs [-0.38, 0.27], R^2^_β_ < .001.

These results indicate that our autobiographical recall procedure did not successfully induce feelings of gratitude over and above a more general positive mood induction. This is an unexpected result since identical procedures revealed strong effects of this manipulation in an earlier study [1]. In the following analyses we took two approaches. First, we used state emotions as continuous predictors to test the association between gratitude and self-control. Supporting the validity of such an approach, one prior investigation also reported that continuous measures of state gratitude—but not happiness—significantly predicted temporal discounting when state emotion was used as a continuous variable [1]. Second, while groups did not differ in self-reported gratitude, we retained Group as a factor to test if metrics other than explicit self-reported emotion (behaviour and ERPs) showed effects of the manipulation.

### Flanker effects

First, we tested the influence of state emotion and our manipulations on the overall flanker effects. Mean correct-trial reaction time (RT) and choice error rates were submitted to factorial MLMs with the within-subject factors of time-point (pre-induction = -1; post-induction = 1) and compatibility (compatible = -1; incompatible = 1). Between subjects variables were continuous ratings of happiness and gratitude in initial models, and subsequent models used the between-subjects factor of group (happiness = -1; gratefulness = 1) without the continuous predictors. Data from two participants (one from each condition) in the pre-induction flanker task was marked as missing because these participants made >50% errors on the initial test (3.4% missing data).

#### Mean RT

We initially used gratitude and happiness as continuous variables in covariate models to predict mean RT. To assess the impact of emotion, we modeled both the two-way interactions between emotion and compatibility (e.g., gratitude X compatibility) and the three-way interactions including time-point (e.g., gratitude X compatibility X time-point). We conducted these analyses rather than conducting a full factorial model as our hypotheses did not refer to main effects of emotion, two-way interactions between emotion and time-point, and interactions between gratitude and happiness.

Response times were longer for incompatible (459 ms) than compatible (397 ms) flanker arrays, confirming the expected flanker effect (*b* = 31.07, *SE* = 1.41), 95% CIs [28.28, 33.86], R^2^_β_ = .75. Participants also responded faster overall on the post-induction (421 ms) compared to the pre-induction (435 ms) task (*b* = -6.84; *SE* = 1.42), 95% CIs [-9.65, -4.03], R^2^_β_ = .12. However, the critical three-way interaction between self-reported gratitude, time-point, and compatibility was not observed (*b* = -0.57, *SE* = 1.47), 95% CIs [-3.47, 2.32], R^2^_β_ < .001. No other main effects or interactions were significant in this model, see Table 1.

**Table 1:** Analyses using gratitude and happiness as continuous predictors of dependent variables.

	Dependent Variable					
	Reaction Time		Choice error rates		ERPs	
*Flanker Effects*	*b*(*SE*)	95% CIs	*b*(*SE*)	95% CIs	*b*(*SE*)	95% CIs
Compatibility	**31.07(1.41)**	**28.28, 33.86**	**4.11(0.28)**	**3.55, 4.67**	-	-
Time-point	**-6.84 (1.42)**	**-9.65, -4.03**	**1.03(0.29)**	**0.47, 1.60**	-	-
Compatibility X Time-point	0.27(1.41)	-2.52, 3.07	0.55(0.28)	-0.008, 1.11	-	-
Compatibility X Gratitude	-0.85(1.47)	-3.75, 2.05	-0.11(0.29)	-0.69, 0.47	-	-
Compatibility X Happiness	-0.36(1.77)	-3.54, 3.47	0.33(0.36)	-0.67, 0.74	-	-
Compatibility X Grat. X Time-point	-0.57(1.47)	-3.47, 2.32	0.34(0.29)	-0.55, 0.62	-	-
Compatibility X Happ. X Time-point	0.88(1.77)	-2.62, 4.38	-0.21(0.36)	-0.92, 0.49	-	-
*Error Adjustments & ERPs*	*b*(*SE*)	95% CIs	*b*(*SE*)	95% CIs	*b*(*SE*)	95% CIs
Trial-type	3.17(2.06)	-0.89, 7.24	**0.89(0.33)**	**0.23, 1.56**	**-4.75(0.27)**	**-5.29, -4.21**
Time-point	**-10.62(2.07)**	**-14.71, -6.52**	**1.14(0.34)**	**0.48, 1.81**	0.42(0.27)	-0.12, 0.97
Trial-type X Time-point	-2.60(2.06)	-6.67, 1.47	0.46(0.33)	-0.20, 1.12	0.34(0.27)	-0.19, 0.88
Trial-type X Gratitude	2.20(2.20)	-2.15, 6.54	0.22(0.36)	-0.49, 0.92	-0.45(0.34)	-1.12, 0.22
Trial-type X Happiness	-5.19(2.64)	-10.40, 0.03	-0.04(0.43)	-0.89, 0.80	0.63(0.42)	-0.20, 1.46
Trial-type X Grat. X Time-point	-2.03(2.20)	-6.37, 2.31	0.004(0.36)	-0.70, 0.71	-0.06, (0.34)	-0.73, 0.61
Trial-type X Happ. X Time-point	4.49(2.64)	-0.72, 9.71	-0.16(0.43)	-1.01, 0.69	-0.35(0.42)	-1.18, 0.48

Sig. effects in bold.

**Fig 2:**
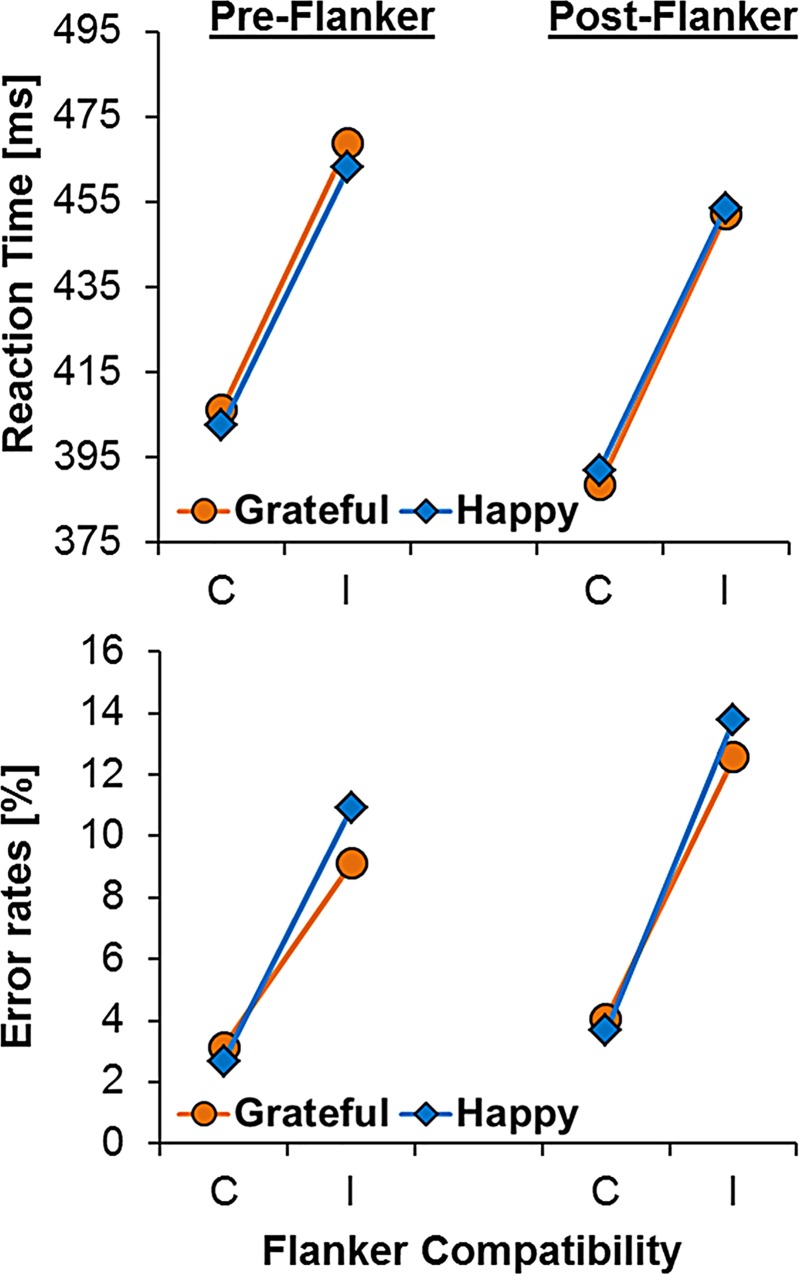
Flanker effects on reaction time (top panel) and error rates (bottom panel) split by induction condition (orange = grateful, blue = happy) and time-point (left = pre-induction, right = post-induction). The x-axis depicts compatibility level: C = compatible trials (e.g., HHHHH); I = incompatible trials (e.g., SSHSS).

In a second model, we replaced the continuous emotion variables with the factor group to represent our experimental manipulation. Here, the critical three way interaction between group, time-point, and compatibility was not statistically significant (*b* = -0.20, *SE* = 11.22), 95% CIs [-21.97, 22.37], R^2^_β_ = .12 (see Fig 2, upper panels). No other novel main effects or interactions were significant in this model, see Table 2 for details.

**Table 2:** Analyses using group as a between-subjects predictor of the dependent variables.

	Dependent Variables					
	Reaction Time		Choice error rates		ERPs	
*Flanker Effects*	*b*(*SE*)	95% CIs	*b*(*SE*)	95% CIs	*b*(*SE*)	95% CIs
Time-point	**16.87 (5.64)**	**5.74, 28.00**	**-3.45 (1.13)**	**-5.68, -1.23**	-	-
Compatibility	**-63.49 (5.56)**	**-74.48, -52.50**	**-8.51 (1.11)**	**-10.71, -6.32**	-	-
Group	1.63 (16.34)	-30.98, 34.24	1.24 (1.56)	-1.85, 4.32	-	-
Compatibility X Time-point	0.98 (7.94)	-14.69, 16.66	2.54 (1.59)	-0.60, 5.67	-	-
Group X Time-point	-7.44 (7.97)	-23.19, 8.30	0.56 (1.59)	-2.58, 3.71	-	-
Group X Compatibility	1.78 (7.87)	-13.76, 17.32	-1.62 (1.57)	-4.73, 1.48	-	-
Group X Compatibility X Time-point	0.20 (11.23)	-21.97, 22.37	-0.64 (2.24)	-5.07, 3.79	-	-
*Error Adjustments & ERPs*	*b*(*SE*)	95% CIs	*b*(*SE*)	95% CIs	*b*(*SE*)	95% CIs
Time-point	**20.78 (8.41)**	**4.17, 37.40**	**-3.43 (1.35)**	**-6.09, -0.77**	-1.69 (1.07)	-3.81, 0.43
Trial-type	-4.48 (8.21)	-20.69, 11.73	**-3.81 (1.32)**	**-6.41, -1.21**	**9.10 (1.05)**	**7.02, 11.18**
Group	-1.77 (17.55)	-36.70, 33.17	-1.44 (1.81)	-5.02, 2.13	1.78 (1.33)	-0.86, 4.41
Trial-type X Time-point	3.04 (11.72)	-20.09, 26.18	2.24 (1.88)	-1.47, 5.94	2.05 (1.50)	-0.92, 5.01
Group X Trial-type	11.14 (11.90)	-12.36, 34.64	0.44 (1.90)	-3.32, 4.20	0.13 (1.50)	-2.84, 3.09
Group X Trial-type	6.62 (11.61)	-16.30, 29.55	2.15 (1.86)	-1.53, 5.82	-0.55 (1.48)	-3.47, 2.37
Group X Trial-type X Time-point	-26.96 (16.57)	-59.69, 5.76	-0.80 (2.66)	-6.04, 4.44	-0.52 (2.11)	-4.68, 3.64

Sig. effects in bold.

#### Choice error rates

Participants made more mistakes for incompatible (11.6%) than compatible (3.4%) trials, indicating the presence of a flanker effect on choice error rates (*b =* 4.11, *SE =* 0.28), 95% CIs [3.55, 4.67], R^2^_β_ = .57. Error rates were also higher at the post-induction (8.5%) compared to pre-induction (6.5%) task (*b* = 1.03, *SE* = 0.29), 95% CIs [0.47, 1.60] R^2^_β_ = .08. As with mean RT, however, the critical three-way interaction between self-reported gratitude, time-point, and compatibility was not significant (*b* = 0.34, *SE* = 0.29), 95% CIs [-0.55, 0.62], R^2^_β_ < .001. No other interactions were statistically significant, see Table 1.

Next we tested the effects of the experimental manipulation of emotion on choice error rates. As with mean RT, the critical three-way interaction between group, time-point and compatibility was not significant (*b* = -0.64, *SE* = 2.24), 95% CIs [-5.07, 3.79], R^2^_β_ = .08, see Fig 2, lower panels. No other effects in this model suggested that our emotion manipulations had any effect on choice error rates, see Table 2. Thus, while we found evidence that flanker performance generally became faster and less accurate between the initial and the second task, we found no evidence that the flanker effect was moderated by state emotions or induction condition.

### Post-error adjustments

We next investigated the association between gratitude and behavioural adaptation following errors. For these analyses we defined two trial-types: trials preceded by a correct trial (post-correct trials) and trials preceded by an error trial (post-error trials). For the RT analyses of the post-error slowing effect, we compared mean reaction times for correct responses on post-correct trials to those for correct responses on post-error trials. Percentage error rates were also calculated for post-error and post-correct trials (e.g., post-error error rate = number of errors on post-error trials / total number of post-error trials * 100). Post-error measurements (RTs and error rates) were marked as missing data if a participant contributed < 6 mistakes to a given RT cell (3.4% missing data). Finally, we only included trials that were preceded by an incompatible trial to avoid an asymmetric contribution of conflict adaptation effects between post-error and post-correct trials (refer to [27] precise details on the data processing for the post-error analyses). The statistical analyses followed those for the flanker effects, with the factor compatibility replaced by trial-type (post-correct = -1, post-error = 1) in all models.

#### Mean RTs

While mean RTs on post-error trials (436 ms) were slightly longer than those on post-correct trials (430 ms), this effect was not statistically significant (*b* = 3.17, *SE* = 2.06), 95% CIs [-0.89, 7.24]), R^2^_β_ = .01. As with the flanker analyses, participants were faster on the post-induction rather than the pre-induction task (*b* = -10.62, *SE* = 2.07), 95% CIs [-14.71, 6.52], R^2^_β_ = .14. However, the critical three-way interaction between trial-type, time, and gratitude levels was also not significant (*b* = -2.03, *SE* = 2.20), 95% CIs [-6.37, 2.31], R^2^_β_ = .005, suggesting post-error performance was not related to gratitude levels. No other interactions were statistically significant, see Table 1.

We next used group as a dichotomous variable to predict RTs, and again found no significant three-way interaction between group, compatibility, and time-point (*b* = -26.96, *SE* = 16.57), 95% CIs [-59.69, 5.76], R^2^_β_ = .02. No other novel main effects or interactions were statistically significant in this model, see Table 2.

#### Choice error rates

Participants made more errors on post-error (8.2%) than post-correct trials (6.4%) (*b* = 0.89, *SE* = 0.33), 95% CIs [0.23, 1.56], R^2^_β_ = .04, suggesting that performance deteriorated on the trials following mistakes. Participants also made more mistakes on the post-induction rather than the pre-induction flanker task, (*b* = 1.14, *SE* = 0.34), 95% CIs [0.48, 1.81], R^2^_β_ = .07. However, as with all other behavioural analyses presented previously, the interaction between gratitude, trial-type, and time-point was not significant (*b* = 0.004, *SE* = 0.36), 95% CIs [-0.70, 0.71], R^2^_β_ < .001. No other main effects or interactions were statistically significant, see Table 1.

We then added group as a between subjects factor to predict error rates. As with other models, we found no additional evidence that post-error adjustments differed between groups, see Table 2. Therefore, while participants became significantly less accurate for post-error compared to post-correct trials, this effect was not associated with gratitude either at the group level, or when we used gratitude scores as a continuous predictor of performance.

### ERP Analyses

To investigate the relationship between gratitude and neural performance monitoring we used an identical model to those used previously where the effect of trial-type represented correct and error related ERPs (CRN = -1; ERN = 1). Five additional participants (2 from the happy condition and 3 from the gratitude condition) were excluded because they made too few errors to calculate reliable ERNs in for both the pre- and post-induction task (< 5, [28]). For the remaining participants, data was scored as missing if participants did not have sufficient usable trials to generate an ERP in a given cell (2.8%).

#### ERN

As expected, ERP amplitudes were significantly more negative for errors (-13.38 μV, *SE =* 0.56) than correct trials (-3.67 μV, *SE =* 0.55) (*b* = -4.83, *SE* = 0.26), 95% CIs [-5.35, -4.32], R^2^_β_ = .70. Contrary to predictions, however, the three-way interaction between trial-type, self-reported levels of gratitude, and time-point was not significant (*b* = -0.13, *SE* = 0.33), 95% CIs [-0.78, 0.51], R^2^_β_ = .001. No other main effects of interactions were significant, see Table 1.

**Fig 3:**
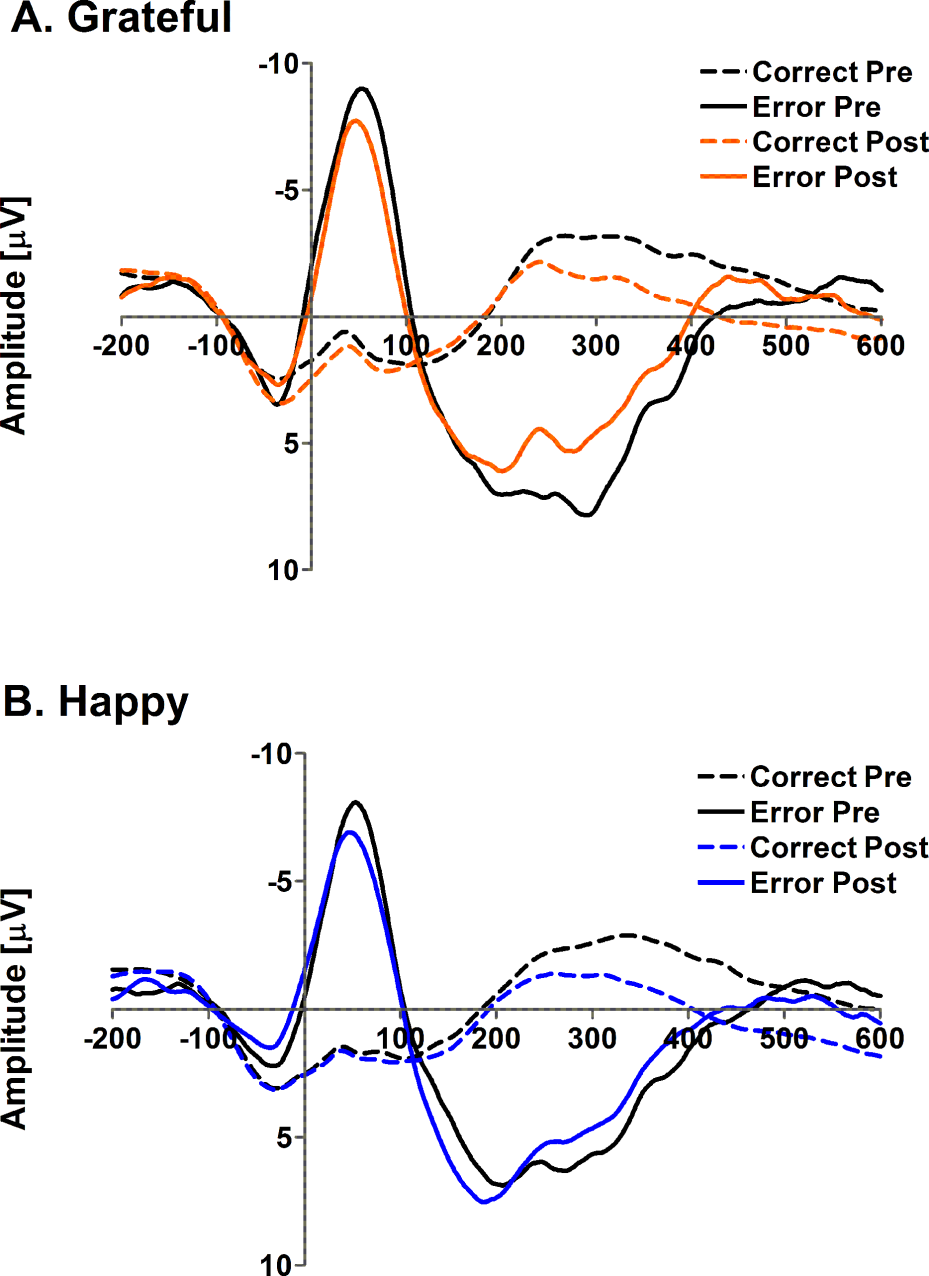
Error-related ERPs split as a function of induction group (top panel = grateful; lower panel = happy), trial-type (correct; error) and time-point (pre-induction = black; post-induction = coloured).

Consistent with all previous models, the three ways interaction between group, trial-type, and time-point was not significant when we added the experimental manipulation as a between subjects factor (*b* = -0.52, *SE* = 2.11), 95% CIs [-4.68, 3.64], R^2^_β_ < .001, see Fig 3. No other main effects or interactions revealed significant effects of the manipulation on ERPs, see Table 2 for details.

Thus, we did not find evidence that gratitude was associated with increased ERN amplitudes either at the group level of analysis or when emotion was used as a continuous predictor.

## Discussion

We investigated the relationship between gratitude and a number of established metrics of self-regulation, including neurophysiological performance monitoring, the ability to override conflict, and post-error performance. Expanding on recent findings [1], we predicted that gratitude would be associated with enhancements across each of these metrics. In contrast to these predictions we found no evidence that gratitude was associated with enhanced self-control when analyses were conducted either at the group level, or using emotion as a continuous predictor of control. The overall picture provided by these results stands in contrast to recent suggestions that gratitude predicts enhanced self-regulation. More specifically, in one prior study increased economic patience was robustly associated with elevated gratitude, operationalized both by experimental induction group (i.e., grateful vs. happy) and when state gratitude was used as a continuous predictor in a regression model [1]. However, our study suggests that analogous effects do not occur in all forms of self-regulation, particularly those observed in established conflict control tasks, such as the flanker paradigm.

Given our results it is important to consider the significance of the current investigation in relation to established associations between self-control and gratitude. Initially, it should be noted that self-control refers to a heterogeneous range of phenomena that are utilized flexibly in a wide array contexts [29–31]. As such, it is perfectly possible that gratitude might influence some—but not all—of the operations that we identify as self-control. In light of this suggestion, it may be the case that gratitude has a rather selective effect on some aspects of self-control (e.g., temporal discounting) while leaving others relatively unaffected (e.g., performance monitoring, conflict control). Rather than challenging prior findings, our results more likely call for a nuanced account where gratitude has a specific—rather than a general—impact on the ability to exert self-regulation.

If this is the case, then, the obvious question arises: Why might gratitude facilitate economic patience yet leave conflict related control adjustments unaffected? First, as specific emotions (e.g., sadness, gratitude) putatively reflect a response to particular challenges [32], it is conceivable that different emotions are more or less relevant for specific self-control functions. Feeling grateful can likely counteract the short-term hedonic costs associated with delayed gratification [1]; individuals who are already experiencing gratitude are—almost by definition—not in the dissatisfied state that putatively fuels preference for immediate rewards. In contrast, emerging evidence indicates that it is actually the negative experience of goal conflict that critically underlies the instantiation of conflict-driven control [18]. Here, aversive conflict experiences motivate the up-regulation of control to avoid similar negative experiences in the future [33–35]. In contrast, incidental positive emotion, such as the sort of incidental gratitude induction we used here, might have little effect on conflict regulation.

Beyond a dimensional view of emotional-cognition integration, there may be utility in studying more discrete emotional categories in the context of conflict control. In a recent correlational study, external punishment, subjective effort, and anxious experience were associated with more accurate performance, while frustration and hopelessness were accompanied with diminished control [36]. Thus, understanding the intricacies of the integration between goal-directed action and emotion may be further enhanced through studying the relationships—for better or worse—between specific emotional experiences and the implementation of self-control.

### Limitations and future directions

The current results should be considered in light of some limitations and potential future research questions. Foremost, despite closely following established induction procedures [1], self-reported gratitude did not differ between recall conditions in our experiment. The source of this discrepancy between studies is unclear as we used instructions identical to those used in the previous study. That said, the failure of this manipulation is a clear limitation of the current results, perhaps also speaking to the efficacy and replicability of this induction protocol more broadly. Nevertheless, previous research also found a relationship between gratitude and self-control when the emotion was used as a continuous predictor [1]. In our study we found no such effect, despite using the same scales used in the prior study.

To date there has been relatively few studies of the relationship between gratitude and self-control. Therefore, if this moral emotion does selectively influence specific aspects of self-control, one direction for future research is to test the boundaries of this effect by assessing impact of gratitude on multiple aspects of self-control. At a low level, this can be achieved by testing the association between feelings of gratefulness and performance on paradigms selected to test specific aspects of control, such as inhibition, updating, goal maintenance, or task switching. In addition to isolating the mechanics of control, however, self-control can also be exerted in an interpersonal context where individuals work simultaneously on a control task [37, 38]. For example, individuals are more reactive to their performance partner’s mistakes if they are performing with cooperative rather than competitive instructions [37]. As gratitude has been particularly related to reciprocal, partner-focused behaviours, the benefits conferred by gratefulness on self-control might be particularly accentuated when control tasks are performed in such a dyadic context.

### Conclusion

In conclusion, we found no evidence that gratitude is associated with increases in a number of self-control phenomena that arise during conflict tasks, including the flanker effect, sequential error adjustments, and neurophysiological performance monitoring. These results suggest that prior benefits of gratitude observed during temporal discounting might not be generalizable to self-regulation more broadly.
